# Patients experience of warmth and coldness in connection with surgery – a phenomenological study

**DOI:** 10.1080/17482631.2020.1858540

**Published:** 2020-12-13

**Authors:** Ingrid L. Gustafsson, Mikael Rask, Kristina Schildmeijer, Carina Elmqvist

**Affiliations:** a Faculty of Health and Life Sciences, Department of Health and Caring Sciences, Centre of Interprofessional Cooporation and Joint Use Whitin Emergency Care (CICE), Linnaeus University, Vaxjo, Sweden; b Faculty of Health and Life Sciences, Department of Health and Caring Sciences, Centre of Interprofessional Cooporation and Joint Use Whitin Emergency Care (CICE), Linnaeus University, Kalmar, Sweden; c Research County Council, Department of Health and Caring Sciences, Centre of Interprofessional Cooporation and Joint Use Whitin Emergency Care (CICE), Linnaeus University, Vaxjo, Sweden

**Keywords:** Comfort, experience, hypothermia, patients, perioperative, reflective lifeworld research, surgery, temperature, warming

## Abstract

**Purpose**: The aim was to describe patients’ lived experience of warmth and coldness in connection with surgery.

**Methods**: A reflective lifeworld research (RLR) approach founded on phenomenology and the methodological principles of openness, flexibility, and bridling were used. The data consisted of 16 in-depth interviews with patients from four hospitals in Sweden.

**Results**: Warmth and coldness in connection with surgery means an expectation to maintain one´s daily life temperature comfort. When patients’ needs of temperature comfort is fulfilled it give a sense of well-being and calmness. Despite the body is covered there are feelings of vulnerability. When patients have the ability to change their own temperature comfort, they feel independent.

**Conclusion**: The individual feeling of temperature comfort could be affected or changed to discomfort during the perioperative context, and an intervention is required to avoid suffering due to the care. An ability to independently influence one´s own temperature comfort can strengthen the patient, whereas the opposite entails suffering in silence. The phenomenon is also related to feelings of confidence about receiving the best care as well as being exposed and vulnerable. When the patient´s need of comfortable temperature is met then feelings of security and sense of well-being emerged.

## Introduction

Perioperative hypothermia is common in connection with surgery, and occurs when core temperature fall below 36°C, a temperature that places patients at risk for several adverse events (Hooper et al., 2009; NICE, 2016; Sessler, 2016). To reduce the risk of hypothermia, health-care professionals should maintain a patient’s normal body temperature by using active and passive warming (Hooper et al., 2009; NICE, 2016). In this article, passive and active warming is termed as heat conservation measures (HCM).

The preoperative phase begins when the decision is made for the patient to undergo surgery (Leinonen & Leino-Kilpi, 1999). During this phase patients’ might sense feelings of worry, loss of control, and vulnerability, especially prior to anaesthesia, making them dependent on staff (Forsberg, Engstrom et al., 2014). In addition, patients may experience anxiety related to something going wrong during or after the surgery (Arakelian et al., 2018; Forsberg, Engstrom et al., 2014; Forsberg, Soderberg et al., 2014). Preoperative anxiety among patients is common (Caumo et al., 2001; Pritchard, 2009b), and an increased level of preoperative anxiety is associated with impaired recovery, a longer hospital stay, and increased levels of postoperative infections (Levandovski et al., 2008). Factors that can affect the patients experience of anxiety include losing control (Arakelian et al., 2018; Pritchard, 2009a; Susleck et al., 2007), an unknown environment (Forsberg, Soderberg et al., 2014; Pritchard, 2009a), and a lack of information about what´s going to happen (Pritchard, 2009a).

The intraoperative phase starts when the patient is transferred to the operation theatre (Leinonen & Leino-Kilpi, 1999). Where the most frequent comment from patients about the operating department environment is that it is cold and that the experience of coldness and chills is the worst aspect of the intraoperative phase (Leinonen et al., 1996). When a patient is transferred to the postoperative unit, the postoperative phase starts. This phase ends when there is no longer the need for nurses to provide care. These three phases—the preoperative phase, the intraoperative phase, and the postoperative phase—comprise the perioperative period (Leinonen & Leino-Kilpi, 1999).

Anaesthesia and surgery present several risks, but one of the most common risk is perioperative hypothermia. According to Eriksson (2018) one of the first human need is to avoid cold, and therefore central to be supported by health-care professionals. During anaesthesia and surgery, patients are unable to meet their own basic human needs, and they require another human to fulfil those needs Eriksson, 2018)during the whole perioperative period. The goal for the health-care professionals is to fulfil these needs and prevent perioperative hypothermia. This can be done with help of evidence-based recommendations, by preventing cooling, providing warming and measuring the temperature to discover the patient’s needs for HCM (Hooper et al., 2009; NICE, 2016). Despite recommendations, many patients do not receive HCM, especially active warming measures as forced air warming before anaesthesia induction and during surgery (Duff et al., 2014; Gustafsson et al., 2017). In Sweden, there are no guidelines about perioperative hypothermia, so the only support the health-care professionals can use are those included in the recommendations to avoid postoperative wound infections in hip and knee replacements (County Councils´ Mutual Insurance, 2015) or recommendations for intraoperative care (Sweden’s Municipalities and Regions, 2019).

Some studies have explored patients’ thermal comfort with the help of scales, and found that patients during the preoperative prewarming, by a self-controlled forced-air gown, report a significantly higher temperature comfort (Benson et al., 2012; Leeth et al., 2010; Wagner et al., 2006). At least one study found that patients report significantly increased level of temperature comfort when they were prewarmed by forced-air blankets (Fossum et al., 2001). While two studies found no significance but increased levels of temperature comfort (Akhtar et al., 2016; Wasfie & Barber, 2015). When preoperative warming is used, patients express a lower level of anxiety (Fossum et al., 2001; Wasfie & Barber, 2015) or they do not experience any differences in their anxiety (Kimberger et al., 2007). In addition, patients experience a more comfortable temperature when disinfectants are preheated rather than at room temperature. Patients who are treated with warm disinfectants want to have the preheated solution the next time disinfectants are required (Wistrand et al., 2015, 2016).

Most studies that have investigated patients’ experiences with temperature are quantitative; to our knowledge, no qualitative studies have been conducted. There is a need for reaching the patients’ insider perspective, through a lifeworld perspective, which can explain the meaning of insiderness (Todres et al., 2014); only they can tell what this inward perspective is (Todres et al., 2009). To close this knowledge gap, there is a need to reach the lived experiences. Therefore, this qualitative study describes warmth and coldness in connection with surgery from the patient’s perspective.

## Materials and methods

### Design

A Reflective Lifeworld Research (RLR) approach, founded on the phenomenology, i.e., the continental philosophy and its philosopher (Dahlberg et al., 2008) was chosen in order to reach the phenomenon, warmth and coldness in connection with surgery. To adopt the RLR approach the methodological principles such as openness, flexibility and bridling was used (Dahlberg et al., 2008), including a reflective approach in order to bridle any natural attitude and not define what is undefined or take the world as it seems to be, for granted (Dahlberg & Dahlberg, 2019).

### Context

Four hospitals were chosen based on the dimensions and number of operating theatres in Sweden, these hospitals have each 6, 15, 15 and 33 operating theatres. The hospitals were also chosen to represent all areas of Sweden: north, south, east and west. In Sweden, hospitals use several types of HCM: cotton gowns, caps, leg covers (quilts shaped as leg sacks), cotton blankets or disposable blankets, quilts, electrical mattresses and forced air warming into gowns, mattresses or blankets, and active-heated intravenous and irrigation fluids.

### Participants and procedures

All directors provided written approval and helped with a contact person at their operating department, whom got information about the inclusion criteria: patients <18 years of age, received neuroaxial blockade and been awake or received a general anaesthesia, been in the operating theatre for at least 1 hour and speak Swedish. Exclusion criteria were cognitive insufficiency, the inability to feel cold or heat because of sickness or syndromes. In addition, patients who received cooling treatment during the surgery were excluded, because their care was out of standard. Patients that met the criteria in the postoperative units were invited to participate. The contact person provided these patients with oral and written information about the study. The patients mailed the signed written consent form to the first author, and got contacted for an interview. In total, 16 patients, nine woman and seven men between 27 and 82 years participated. Four patients had day surgery, eight stayed overnight in a hospital ward or patient hotel, and four were in-patients. The surgeries were surgical, orthopaedic and gynaecological.

### Data-collection

The interviews were open-ended to obtain in-depth information about the lived experiences of warmth and coldness in connection with surgery. The patients chose where the interview was conducted: nine interviews in the patients’ own homes; two at their workplaces (free from work); two in a café; one at a restaurant; one in a patient hotel room; and one by Skype™ from the patient´s workplace. To reach the phenomenon, the interviews started with an open-ended question: Can you tell me about your experiences in connection with your surgery? To reach deeper into the phenomenon, a more direct question was asked: When you were waiting in the preoperative unit, how did you experiences warmth and coldness? Follow-up questions were also asked: e.g., You told me that when you were given a warm blanket it felt warmth and as to be embraced, could you expand on that more? Can you further describe that feeling of coldness? The interviews lasted between 18 and 53 minutes (average 32 min) and were recorded and transcribed verbatim by the first author. Data were collected between January 2017 to February 2018.

### Data-analysis

In a phenomenological analysis, the goal is to search for and describe the essence of the phenomenon. The analysis entails a flexible movement between the whole, the parts and the whole to reach abstraction of the lived experience. It is important to understand each parts in terms of the whole and understand the whole in terms of its parts. The analysis process, following the principles of RLRs approach (Dahlberg et al., 2008), started by reading each interview several times to become familiar with the data and to gain a sense “of the whole” of the text. With the phenomenon “warmth and coldness in connection with surgery” in mind, parts of the text related to the phenomenon was divided into meaning units. Meanings within every meaning unit were sought for and was discussed and reflected upon by the first and last author in order to bridle the pre-understanding during the analysis. The meanings were then grouped into patterns according to similarities and differences by using part of the patterns figuratively and parts as background reasoning, to find more or less hidden meanings in the phenomenon. The patterns of meanings were combined into clusters on an abstract level and then related to each other to find the essential meaning structure of the phenomenon—i.e., its essence. All authors have reflected on and discussed the findings in a flexible movement between whole, part and whole. The findings are presented as an essence followed by constituents with quotations from the data to describe the variations of the phenomenon.

### Ethical considerations

Ethical approval was granted by the Regional Ethical Comittee, Linkoping, Sweden (Dnr 2016/223-31) and the study was designed to be in line with the Helsinki Declaration (World Medical Association, 2013). The informed written consent explained that patients could withdraw from the study whenever they wanted without explanation. All interviews were coded to guarantee patient confidentiality. The study’s benefit of increasing knowledge about patients’ need for warmth and coldness in connection with surgery is higher than the risk presented by participating in the study.

## Results

The essence of the phenomenon of warmth and coldness in connection with surgery means confidence in receiving the best care together with an expectation to maintain one’s own unique temperature comfort. Warmth and coldness is variable over time and space, where the memory is more or less present. Temperature discomfort requires interventions to prevent suffering from care. Exposure and vulnerability in being undressed exists although heat conservation measures of covering the body is performed. The ability to independently influence one’s own temperature comfort is strengthening, whereas the opposite means suffering in silence. When the need of a comfortable temperature is met, well-being and calmness emerges i.e., being embraced.

### The span between freezing and being too warm

Warmth and coldness in connection with surgery means a span between freezing and being too warm, a dimension that can constantly be changeable. It is unique and stands in contrast to the body’s normal experience of temperature in daily life. There are feelings of always freezing, being neutral or always being too warm. On the one hand, there is a sensitivity to changes in temperature, while on the other hand there is flexibility. In connection with surgery, warmth and coldness are experienced as different from daily life. A new need may arise which previously did not exist, and the need may change over time. *“I felt // so warm and cozy because I don’t usually like that because I usually don’t like it when it is warm // remember when I woke up // … then it was not cozy to be awake [laughter] but then I wanted it cool again as I usually do”* (P3). The body’s experience of being comfortable means feeling neither cold nor sweaty, but being neutral and comfortable. What is comfortable differs for each individual and can be problematic to explain. On the other hand, being too warm feels unpleasant and requires a change, though not as immediate as when a patient freezes. The surrounding environment is experienced as cool yet pleasant, both experiences create a sense of security and give a sense of being accommodated. When the surrounding environment changes, it emerges a sense of cold, that turn into a chilliness that is stronger than normal and increases the longer one waits. The temperature in the operating theatre varies from comfortable to being very cold. *“you come into the operating theatre and it is very very cold ah // oh what can it be … 14 degrees in the air. or it feels as if you were coming into an air … like an air-conditioned room that is far too cold”* (P16). In the body, different body parts can also be more sensitive than others and that can generate a feeling of cold throughout the whole body. A sensation of frozenness can come later in the perioperative process and can occur when one awakens. Likewise, a new sense of being cold may occurs when arriving home, which differs from one’s normal unique temperature.

### Covered but still undressed

Warmth and coldness in connection with surgery means that integrity is restricted, the lived body is experienced as vulnerable and one’s identity disappears in connection with changing out of one’s clothes. Gaining heat conservation measures creates a feeling that the vulnerable body is covered up and protected, despite there being a feeling of exposure and loss of identity. Vulnerability occurs when it is not possible to wear the amount or shape of clothing that is customary in everyday life. *“then you feel that you are wearing less, these big nightshirts they are so airy so you feel naked though you have something on you”* (P5).

Hospital clothing is experienced as something unnerving, necessary and required, there is an acceptance that it needs to be practical, wherein they experiences it as comfortable and isolating against the cold. Heat conservation measures can be cold, thin and flowing instead of being close to the body. *“And, there’s a big differences in only getting this cotton blanket, partially because it is too thin in a way and it can’t create that heat”* (P1). On the other hand, they can be experienced as heavy, light and big, with more volume, which gives a sense of luxury. When changing back to one’s own clothes again, a good feeling emerges together with a sense of relief, and the feeling of being ill disappears.

### Striving to maintain normal body temperature

Warmth and coldness in connection with surgery means striving to maintain one’s own normal temperature comfort. Associated with this, there is also an expectation that the health-care professionals will resolve the unique needs. The exposure and vulnerability in the perioperative environment are depicted as being limiting to one’s independence. If there is no freedom of choice available to access heat, the patient experiences a need to physically move to try to reduce the experience of freezing. Alternatively, try to relax in an attempt to endure and to have patience with the cold. Both situations involve suffering. The dependency affects patients, they may not dare to ask for help with heat conservation measures if their bodies freezes. *“ but I froze so much or oh you know so your teeth chatter but you know then you don’t want to intrude either”* (P9). On the other hand, there is a belief that if they were to ask the health-care professionals for more heat, they would get it. If they dare to ask and the health-care professionals forget or do not see that such needs exist, it is experienced as suffering. In contrast, when feeling too warm, it is easier to ask for help. Being able to independently influence one’s own comfortable temperature is of great importance, and they attempt to correct their own temperature, through their own measures, if possible. *“so that quilt became too much so I scrunched it together and folded it down so it lay at my feet only yeah my feet I had actually [laughter] gently moved them outside so that I had my toes as cooling flanges”* (P4). An internal process is ongoing about the consequences of taking on or taking off layers, a consideration of how it would be experienced before the decision is made. A feeling of independence arises when freedom of choice is created through the staff´s questions about how one’s body temperature is experienced.

### A conscious and subconscious non-prioritization of warmth and coldness

Warmth and coldness in connection with surgery means a priority that can be downplayed through knowledge. The cool operating theatre conjures up a sense of cleanliness and a belief that it is positive for health, and one adapts to the event. *“it would have been worse if you’d come into a warm room with contagions, // I associate warmth with contagions, yeah, so when it is cool I think for me anyway it is a psychological effect also, that it feels clean and fresh”* (P12). At the same time, there is knowledge that they should receive heat conservation measures. *“I felt for, myself, the mattress, so that it was warm and like this just because of the fact that the body temperature drops when you are anesthetized”* (P2). Experiences of warmth and coldness, can be downplayed when the subjective body senses other strong experiences as when much is happening in the operating theatre that affects the patient’s focus. On the one hand, priority is given to not freeze over being too warm. On the other hand, the worst feeling is that of sweating, even though the feeling of being cold is also negative. The feeling of frozenness, can also be suppressed. When there is insight that the operation will start, and soon can be placed behind one, the memory of warmth and coldness is set aside. The same insight, or knowing the cause of the cold, can give the patience needed to deal with the cold. Even discomfort from heat conservation measures like tightness against the body, can be accepted more readily than being cold. When awakening after surgery, a change from unconsciousness too consciousness occurs, and thereby the memory is experienced as diffused. *“I really awoke a little // and then suddenly oh God how hot it is, then I realized somehow what is wrong it feels like something is wrong in my body somehow”* (P3). When the memory of warmth and coldness are missing afterwards, it is interpreted as though no negative experience occurred to give any lasting memory. However, the clearest memories appear to be those where heat conservation measures gave security or where there was a lack of heat conservation measures in previous operations. There is a need to convey one´s feelings of what is experienced in order to shed light on the lack of heat conservation measures. Experiences of coldness in connection with previous operations can prepare for how it is experienced next time.

### From suffering to be-embraced when one´s needs are fulfilled

Warmth and coldness in connection with surgery means a feeling of suffering when needs are not fulfilled, and a feeling of being cherished and embraced when they are. It’s unpleasant when the need for warmth is not filled, a feeling of worry appears, focus is moved to oneself, and it is experienced as being introverted. The body reacts by becoming tense as well as stiff, and to freeze and shivery is experienced as exhausting for the body and is perceived as horrible and something that demands a quick change. A sensation of care arises when health-care professionals ask about the patient’s temperature or pay attention to their need for warmth. When health-care professionals see the needs of the unique human being, there is a sense of security, with the patient feeling courage to hand over the care of their body and to feel ready for what is to come. *“you hand over your body to another person … to get that warmth maybe is needed, that it is perhaps an asset”* (P14). The same feelings are experienced when health-care professionals show an awareness of cold environments and give more heat conservation measures. Even a health-care professional’s emotional approach is experienced as warmth and conveys the same dignity as having one’s physical needs satisfied. When the heated material is placed on the body, a feeling of home occurs. *“I felt very much, you know, you are lying under your cuddly quilt at home // yeah it really felt so nice really so entirely”* (P1). Furthermore, when the heat conservation measures are tucked in around the patient, it is experienced as protective and as being taken care of. The calm that emerges from warmth creates an experience of the brain being more logical and the body more easily controlled. The embracing warmth from heat conservation measures gives a pleasant feeling that creates well-being that makes the body relax and a sense of security arise.

## Discussion

In the present study, it was important for the patients to get care from an individual perspective, to have their own unique temperature comfort in connection with surgery. However, the normal need of warmth and coldness could change, due to the perioperative context. The environments influence were sensed as cold/chilliness in relation to draught and cold air in the operating department. This experience is consistent with the International Organization for Standardization (ISO) conclusion that thermal sensations are related to the thermal balance of the body as a whole and can be affected by cloths, physical activity, and environmental factors such as air movement, temperature, and humidity. When people feel too cold or too warm in a given environment, they experience discomfort via unwanted local cooling/heating such as a draughts, vertical air temperature differences, and cold/warm surfaces (ISO, 7730:^2005^). Temperature comfort/discomfort is associated with skin temperature (Candas & Dufour, 2007) and a comfort zone between 31°C and 34°C (Auliciems & Szokolay, 2007). In this study, some body parts of the patients were more sensitive than other body parts to temperature. For example, most the patients identified their feet as the most sensitive body part. Localized temperature discomfort, such as cold feet, had a large impact on their whole body comfort. According to Mishra, Loomans and Hensen the most sensitive body parts, the hands and the legs, influence the overall sensation in a cold situation (Mishra et al., 2016). Persons feeling cold in their whole body prefer to have warm feet (Zhang et al., 2010). Due to a lower blood flow, women often have lower hand and toe skin temperatures than men (Candas & Dufour, 2007). Furthermore, woman prefers a warmer environment because of higher sensitivity to temperatures and higher comfortable temperature levels (Lan et al., 2008). However, in the present study, both the woman and men with cold feet sensed discomfort. Surprisingly, the sense of temperature can be a low priority relative to other strong subjective feelings and the effects of anaesthesia, unaware of temperature senses. Temperature comfort is individual, and normal boundaries can shift when in preoperative settings, and sense of temperature comfort can be low prioritized. However, if the patient has a cold body part, it can lead to an overall sense of being cold.

In the perioperative context, patients often experience feelings of being exposed and vulnerable. In this study, participant noted that although they had HCM such as clothes and blankets that cover their body, they experienced feelings of vulnerability and loss their integrity. The hospital clothes felt unaccustomed, required, and not shaped as their daily life clothes, which creates a feeling of being naked and exposed. Similar results are seen in earlier studies, where patients felt exposed and not decent in hospital clothing, due to the poor fit of the clothes, especially as the result of the bad fittings and slits in the back of the theatre gowns (Baillie, 2009; Forsberg, Soderberg et al., 2014; Matiti & Trorey, 2008). Additionally, patients can also experience a sense of exposure due to the environment (Widäng et al., 2008). However, patients in the present study expressed the insight that it is not changeable due to the hospital settings. This is similar to the findings in Baillie, where patients experienced the hospital gown as a norm to wear (Baillie, 2009). Depending on routines and environments during their care, patients make changes to their boundaries to manage what is happening (Widäng et al., 2008). Patients’ beliefs and knowledge about temperatures in the perioperative context can influence how they view their needs and priorities. In the present study, patients thought that a cold environment was good for their health or they had knowledge about the importance of not losing heat. The patient lacked information about the importance of feeling normal and warm in connection with surgery; this lack of information can result in unnecessary suffering. Health-care professionals are obliged to give patients information to increase their independency and participation in their care (ICN, 2012; Patient Safety Act, 36:569) and medical treatment (ICN, 2012). To fulfil this, and to support patients in their health process the operation departments and operating clinics need to provide information about the importance of avoiding heat loss during the perioperative period.

In the present study, patients striving to maintain their normal temperature comfort and relying on health-care professionals’ actions can be dependent or independent. In cases where patients do not have the possibility to control their own needs of HCM, they feel limited in their independence. According to Lindwall this change from independence to dependence can happen very quickly in perioperative settings (Lindwall, 2004). To not jeopardize their relationship with their health-care professionals, patients do not complain or do as they are told in an attempt to maintain their dignity and gain the respect of their health-care professionals (Baillie, 2009). In the present study, the patients did not dare ask for more HCM when they were cold. Instead, they coped with the situation by relaxing or physically moving in an attempt to warm themselves. However, if the health-care professionals had asked for their temperature comfort, they believed that they would get HCM. Forsberg et al. had similar results: if health-care professionals ask the patient, they feel as they are allowed to mention if they are uncomfortable (Forsberg, Engstrom et al., 2014). A good way to reach patients’ needs in connection with surgery is through perioperative dialogue, where the patients can express their expectations, thoughts, and stories and where perioperative nurses can support and give them information (Lindwall et al., 2003). In cases where patients in the present study had a freedom of choice or the opportunity to give an answer about their temperature comfort, they could influence their own temperature, making them more comfortable and giving them control over something. By paying attention to the patients’ needs for integrity and dignity, by encouraging them to ask for what they need, and by asking patients about their temperature comfort, health-care professionals can give their patients the possibility of controlling their temperatures by themselves.

In the present study, when patients’ needs were met, their feelings shifted from suffering to feelings of being embraced. When the heated HCM was put on, they sensed a feeling of home. Furthermore, they feel protected, when they were tucked in with blankets/quilts. This warmth embraces them and they feel a sense of well-being, relaxing their bodies, and they felt secured in handing themselves over to the health-care professionals. These findings are supported by studies at a prehospital setting, where patients felt secure, pleasant, and relaxed on a warm mattress (Alex, Karlsson & Saveman, 2014), and felt that the heat spread to their entire body when using warming pads (Alex et al., 2013). Furthermore, prewarming in operation departments using forced air shirts/blankets lowered anxiety in patients (Fossum et al., 2001; Wasfie & Barber, 2015). Perhaps, the accompanying feelings of security, relaxation, and the sense of being home can explain why anxiety level decreased. Even the health-care professionals’ emotional approach gave them a sense of warmth, which was stated as equally important as their physical needs. To reduce the feelings of being vulnerable, dependent, and suffering, health-care professionals need to reach the patients´ “insiderness” by asking for and listening to their lifeworld “the view of the other” (Todres et al., 2014). The patients need of feeling temperature comfortable, relaxed and open to handing themselves over to what is awaiting them is a health process that can be supported by health-care professionals.

### Strengths and limitations

The included patients varied in gender, age, and type of surgical intervention, day and/or in-hospital surgery, which gives a rich variation of the phenomenon. The in-depth interviews were rich on meanings. Fourteen of the patients received general anaesthesia and two received regional anaesthesia. These latter two had been awake for a longer period in the operating theatre before given deep sedation. However, no patient was awake during the whole surgery, which could be seen as a limitation of the study.

As a researcher, there is always in the owns´ lifeworld an understanding, as human is always directed towards something by the intentionality, and thus an object is experienced as it is or as something and a total objectivity can never be (Van Wijngaarden et al., 2017). Therefore, it is important to maintain the RLR´s principles of openness, flexibility and bridling during the interviews and the whole analysis process (Dahlberg et al., 2008). By reflecting during the interviews, asking clarifying questions and not take anything for granted, the first author strived for openness and bridling. During the analysis process, the authors bridled their natural attitude, by an openness to the phenomenon, closeness to the text, and reflection in the group of authors, to not decide what is undefined, remaining as objective as possible. The first author analysed the result in consultation with the other authors, manly with the last author. The results were reflected upon and critically reviewed by other researchers at seminars, which increased objectivity and validity. When the phenomenon is described on a general abstract level and the research is meaning-oriented, phenomenological research is seen as valid (Van Wijngaarden et al., 2017). Variation of the phenomenon together with a carefully described analysis increase the validity of the findings. The results are presented at a general abstract level, which means that “warmth and coldness” could be transferable to similar contexts within hospital settings.

## Conclusion

An individual feeling of temperature comfort could be affected or changed to a feeling of discomfort during the perioperative period due to a cold environment and a draught. An intervention is required to avoid suffering due to the care. The ability to independently influence one´s one temperature comfort can strengthen the individual, whereas the opposite entails suffering in silence. The phenomenon of warmth and coldness in connection with surgery is, however, not just about temperature comfort but is also related to feelings of confidence about receiving the best care as well as being exposed and vulnerable. It is important that when the patient´s need of a comfortable temperature is met then feelings of security and being embraced were generated together with a sense of calmness and well-being.

## Clinical implications

In order to meet the patients’ needs and to strengthen and support their health processes, health-care professionals need to ask and carefully listen to the patients in order to reach their “insiderness” and grasp their unique needs of temperature comfort during the perioperative period. Furthermore, health-care professionals should informed the patients about the importance of maintaining normal body temperature during the whole perioperative period. Moreover, it is important to have an awareness that one cold body part can influence the overall feeling of being cold in the whole body. If patients have some body parts that are more sensitive than other body parts, health-care professionals should provide more HCMs to these body parts early in the process. Furthermore, health-care professionals should give patients the possibility to control their temperature comfort by themselves by having extra HCMs nearby and encouraging their patients to express when they are too cold or too warm. By gaining more knowledge about the patients’ experiences, health-care professionals can reach an understanding of the patients’ points of view, and be able to fulfil their needs and provide care. More knowledge can also increase patients’ participation in their perioperative care. Future research is needed to describe HCMs from different health-care professionals’ perspectives.
